# The Role of Laparoscopy and Ultrasonography in Pancreatic Head Carcinoma

**DOI:** 10.1155/1997/72893

**Published:** 1997

**Authors:** Hans G. Beger, Michael H. Schoenberg

**Affiliations:** University Hospital of Surgery Steinhoevelstrasse 9 Ulm 89075 Germany

## Abstract

*Objective*: The authors performed a prospective evaluation of staging laparoscopy with laparoscopic ultrasonography in predicting surgical resectability in patients with carcinomas of the pancreatic head and periampullary region.

*Summary Background Data*: Pancreatic resection with curative intent is possible in a select minority of patients who have carcinomas of the pancreatic head and periampullary region. Patient selection is important to plan appropriate therapy and avoid unnecessary laparotomy in patients with unresectable disease. Laparoscopic ultrasonography is a novel technique that combines the proven benefits of staging laparoscopy with high resolution intraoperative ultrasound of the liver and pancreas, but which has yet to be evaluated critically in the staging of pancreatic malignancy.

*Methods*: A cohort of 40 consecutive patients referred to a tertiary referral center and with a diagnosis of potentially resectable pancreatic or periampullary cancer underwent staging laparoscopy with laparoscopic ultrasonography. The diagnostic accuracy of staging laparoscopy alone and in conjunction with laparoscopic ultrasonography was evaluated in predicting tumor resectability (absence of peritoneal or liver metastases; absence of malignant regional lymphadenopathy; tumor confined to pancreatic head or periampullary region).

*Results*: “Occult” metastatic lesions were demonstrated by staging laparoscopy in 14 patients (35%). Laparoscopic ultrasonography demonstrated factors confirming unresectable tumor in 23 patients (59%), provided staging information in addition to that of laparoscopy alone in 20 patients (53%), and changed the decision regarding tumor resectability in 10 patients (25%). Staging laparoscopy with laparoscopic ultrasonography was more specific and accurate in predicting tumor resectability than laparoscopy alone (88% and 89% versus 50% and 65%, respectively).

*Conclusions*: Staging laparoscopy is indispensable in the detection of “occult” intraabdominal metastases. Laparoscopic ultrasonography improves the accuracy of laparoscopic staging in patients with potentially resectable pancreatic and periampullary carcinomas.


### THE ROLE OF LAPAROSCOPY AND ULTRASONOGRAPHY IN PANCREATIC HEAD CARCINOMA

### ABSTRACT

*John, T.G., Greig, J.D., Carter, D.C. and Garden, O.J. (1995) Carcinoma of the pancreatic head and periampullary region. Annals of Surgery; 221: 156–164.*

***Objective*: The authors performed a prospective evaluation of staging laparoscopy with laparoscopic ultrasonography in predicting surgical resectability in patients with carcinomas of the pancreatic head and periampullary region.**
***Summary Background Data*: Pancreatic resection with curative intent is possible in a select minority of patients who have carcinomas of the pancreatic head and periampullary region. Patient selection is important to plan appropriate therapy and avoid unnecessary laparotomy in patients with unresectable disease. Laparoscopic ultrasonography is a novel technique that combines the proven benefits of staging laparoscopy with high resolution intraoperative ultrasound of the liver and pancreas, but which has yet to be evaluated critically in the staging of pancreatic malignancy.**
***Methods*: A cohort of 40 consecutive patients referred to a tertiary referral center and with a diagnosis of potentially resectable pancreatic or periampullary cancer underwent staging laparoscopy with laparoscopic ultrasonography. The diagnostic accuracy of staging laparoscopy alone and in conjunction with laparoscopic ultrasonography was evaluated in predicting tumor resectability (absence of peritoneal or liver metastases; absence of malignant regional lymphadenopathy; tumor confined to pancreatic head or periampullary region).**
***Results*: "Occult" metastatic lesions were demonstrated by staging laparoscopy in 14 patients (35%). Laparoscopic ultrasonography demonstrated factors confirming unresectable tumor in 23 patients (59%), provided staging information in addition to that of laparoscopy alone in 20 patients (53%), and changed the decision regarding tumor resectability in 10 patients (25%). Staging laparoscopy with laparoscopic ultrasonography was more specific and accurate in predicting tumor resectability than laparoscopy alone (88% and 89% *versus* 50% and 65%, respectively).**
***Conclusions*: Staging laparoscopy is indispensable in the detection of "occult" intra-abdominal metastases. Laparoscopic ultrasonography improves the accuracy of laparoscopic staging in patients with potentially resectable pancreatic and periampullary carcinomas.**

KEY WORDS: Pancreatic carcinoma laparoscopy ultrasonography

### PAPER DISCUSSION

The incidence of the pancreatic carcinoma has increased in the last years and malignant lesions of the pancreas, especially of the pancreatic head and periampullary region, constitute a major problem in surgical practice. Most patients exhibit local invasion of the tumor and metastatic spread by the time symptoms occur. Nevertheless, fortunately, the partial duodeno-pancreatectomy (Whipple procedure) can be performed safely with a low morbidity and mortality rate[1]. Although surgical resection of the tumor is often the only prospect for prolonged survival if the tumor can be removed entirely, resectability rates still remain rather low. They do not exceed over 30% of all patients suffering from malignancy in the pancreatic gland, although the rate of resection has been increasing in the last years.

The low resectability rates are due to local invasion of the tumor into adjacent structures, such as vessels, duodenum, gastric bowel and distant metastases. The question therefore remains how to avoid "non-therapeutic" needless laparotomy in these patients by safely assessing resectability preoperatively with simple non-invasive means.

Unfortunately, the pancreatic gland is difficult to assess by imaging procedures due to its anatomic localization. Consequently, various imaging procedures, invasive, non-invasive or semi-invasive have been employed. Among them ultrasonography, CT-scan and ERCP have proven to be useful, repeatable and relatively inexpensive. However, all of these procedures have their limitations especially concerning not so much the diagnosis of pancreatic carcinoma but its surgical resectability. All too often in the past, patients underwent laparotomy for pancreas tumor and were found to be irresectable.

We and many of the surgeons believe that selective coeliac angiography is a valuable tool in defining locally advanced tumors as demonstrated by infiltration, stenosis or occlusion of the extrapancreatic arteries and veins. Besides predicting tumor unresectability it also provides information about possible vasculur anomalies which are present in about one third of the patients.

Recently, several new, more invasive methods for diagnosis and assessment of pancreatic tumors have been described. Among them, endoscopic ultrasonography is able to assess the size of the tumor, thus tumor stage and the lymph node status. It has been shown that the overall accuracy is high[2].

The purpose of this study was to assess (in a prospective evaluation) staging of the pancreatic carcinoma by laparoscopy and *laparoscopic* ultrasonography. In this study, 40 patients diagnosed as having pancreatic or periampullary carcinoma, were considered as candidates for tumor resection. Already in the referral hospitals, various diagnostic procedures were performed in order to ensure by ERCP, dynamic CT-scanning, sonography, that the tumor would be resectable. The authors are aware that this highly selective group of patients introduces a certain element of bias, however, it mirrors the clinical situation of a specialized clinic into which these patients are referred to after initial diagnosis. The results show that laparoscopy, a rather invasive method for assessing resectability of the pancreatic tumor, is only reliable if a "positive" result is obtained. In this study, laparoscopy failed to identify local or regional tumor growth in twelve patients which later on was found intraoperatively. This results in a specificity of 50% and an overall accuracy of 65%. The importance of the method is if during laparoscopy in combination with biopsy a "positive" results can be obtained. The same holds true for many other methods, such as colonoscopy, where only the positive results that is proven malignancy is of true diagnostic value. Negative results do not exclude malignancy or spread of the tumor.

In contrast, laparoscopic ultrasonography showed an extreme high specificity and accuracy of 88 and 89%. If one however critically reviews the results, the authors have not compaired this rather invasive method with the less invasive methods such as endosonography. Moreover, the authors did not open the lesser sac which by laparoscopic means can be done fast and safely, unless the patient has previously suffered from other pancreatic diseases, such as chronic pancreatitis. The authors found that laparoscopic ultrasonography altered the staging information of the patients, thus the decision concerning tumor resectability in 10 patients (25%). From these 10 patients, however, 8 had invasion of the adjacent superior mesenteric and portal vein. If the authors had performed an angiography prior to an invasive procedure, such as laparoscopy and laparoscopic ultrasonography, they most likely would have also found these alterations excluding resectability. Therefore, this expensive invasive method was of benefit only in 2 out of 40 patients. It remains thereforequestionable if in a large group of patients this expensive and invasive method really adds significantly to the information necessary to decide whether a tumor is resectable or not.

The methods described in this study, considering their limitations and possibilities, are ethically only feasible if they are used just prior to laparotomy. They might help to avoid unnecessary laparotomies, however, often although unresectable, the surgeon will perform bypass surgery according to the prognosis of the tumor. Therefore, in doubtful cases one should decide for the operation since it's the only treatment with any curative chance.

Our concept concerning assessment of resectability includes in the first line the diagnosis of the pancreatic tumor. The imaging procedures include conventional ultrasonography, dynamic CT-scan, and ERCP. Thereafter, unless already unresectability is proven by the imaging procedures an angiography is performed in order to assess the spread of the tumor into the vessels and/or possible vascular anomalities. Lately, this imaging procedure has been substituted in selective cases by endoscopic ultrasonography. In our experience, this new imaging procedure can be used for tumor growth assessment and also infiltration of the vessels adjacent to the pancreas. This method, in experienced hands, reaches a sensitivity and specificity of nearly 90%.

In doubtful cases and in patients with high perioperative risk, a laparoscopy has been performed and in most cases the lesser sac was opened and inspected. With this procedure, we were able to decrease the number of unnecessary laparotomies significantly. In times of cost efficiency and limited resources new diagnostic measures have to be tested against non-invasive cheaper methods. As stated also by the authors, these new methods have to be evaluated against conventional investigations in prospective comparative studies.

### REFERENCES

1. Trede, M., Schwall, G. and Saeger, H.D. (1990) Survival after pancreatoduodenectomy: 118 consecutive resections without an operative mortality. *Ann Surg*; **211**, 447–458.
2. Rösch, T., Braig, C. and Gain, T. *et al.* (1992) Staging of pancreatic and ampullary carcinoma by endoscopic ultrasonography: comparison with conventional sonography, computed tomography, and angiography. *Gastroenterology*; **102**, 188–199.

Hans G Beger, MD
Professor of Surgery, FACS
Head of Department of Surgery
Michael H. Schoenberg MD
Associate Professor
University Hospital of Surgery
Steinhoevelstrasse 9
89075 Ulm
GERMANY

### OPERATIVE VS NON-OPERATIVE MANAGEMENT IN STERILE NECROTIZING PANCREATITIS

### ABSTRACT

*Rau, B., Pralle, U., Uhl, W., Schoenberg, M.H. and Beger, H.G. (1995) Management of sterile necrosis in instances of severe acute pancreatitis. Journal of The American College of Surgeons, **181**: 279–288.*

***Background:*** **The clinical management of sterile pancreatic necrosis is still a matter of debate. In this study we analyzed the clinical course and outcome of patients with sterile necrotizing pancreatitis treated surgically versus nonsurgically.**
***Study Design:*** **Between May 1982 and December 1993, 249 patients with necrotizing pancreatitis (NP) entered this study, of which 172 (69 percent) had intraoperatively or fine needle aspiration-proven sterile NP. One hundred seven of 172 patients underwent surgery (S group) with necrosectomy and continuous postoperative closed lavage and 65 of 172 were treated by nonsurgical means (NS group).**
***Results:*** **Median Ranson and admission APACHE II scores were 4.7 (range, 1 to 10) and 11 (range, 1 to 29) in the S group, significantly higher than those in the NS group with 3.0 (range, 0 to 6) (*p*=0.022) and 8 (range, 1 to 23) (*p*=0.036). After 48 hours of intensive care treatment, APACHE II scores persisted at 10.5 (range, 1 to 29) in the S group and decreased to 6 (range, 0 to 15) (*p*=0.013) in the NS patients. Median C-reactive protein (CRP) levels on admission were 179 mg/L and 68.5 mg/L (*p*=0.023), respectively. Within 72 hours, 61 (94 percent) of 65 NS-managed patients responded to intensive care therapy, whereas organ complications persisted or increased and thus led to surgery in the S group. Mortality rates were 13.1 percent in the surgically treated patients and 6.2 percent in the nonsurgically treated patients (*p*=NS).**
***Conclusions:*** **Most patients with limited and sterile pancreatic necrosis respond to intensive care treatment. Indication for surgery in sterile NP should be based on**

